# Inflammation of the Parotid Glands

**Published:** 1860-11

**Authors:** Charles Brackett


### ARTIC E 4.

### INFLAMMATION OF THE PAROTID GLANDS.

BY CHARLES BRACKETT, M. D.

Sunday, August 26, 1860.—Mr. Sellers, of Marshall Co., brought to me his boy, aged about six years. A bright boy in all respects. Scrofulous diathesis; blue eyes, light hair; firm globular tumor under each ear, reaching forward, of the size of a hen's egg; had suffered; does yet cause some pain; feverish by turn. Glands inflamed about ten days; had been treated or prescribed for by Drs. Manneville and Wertz. I gave morphine when necessary to procure rest. Applications of warm water dressings, stramonium leaves, &c., and directed iod. if suppuration did not take place in a few days. The tumors were hard, skin not discolored, firm, and with no appearance of suppuration.

On Sunday night, after their return home, which is some twelve miles from here, they sent the boy to bed as well as usual, except some fatigue from his long ride. About midnight he made some noise, and awakened the family by his cries. After a few minutes he appeared better. Soon again he appeared to suffer, with considerable dyspnœa. They then sent for Dr. Wertz, living not far off. Before he came, the boy was easy again. Soon after his arrival, he was again suffering; matter poured out of his nostrils and mouth. He become easy again, and his father thought his danger was all past; took him in his arms, the boy breathiug easily; became suddenly pale and expired without a struggle. Why did he die or how?

